# Long Non-coding RNA in CNS Injuries: A New Target for Therapeutic Intervention

**DOI:** 10.1016/j.omtn.2019.07.013

**Published:** 2019-07-29

**Authors:** Li Zhang, Handong Wang

**Affiliations:** 1 Department of Neurosurgery, Jinling Hospital, School of Medicine, Nanjing University, Nanjing, Jiangsu Province, China

**Keywords:** CNS injuries, long non-coding RNAs, MALAT1, MEG3, downstream molecules

## Abstract

CNS injuries, such as traumatic brain injury (TBI), subarachnoid hemorrhage (SAH), intracerebral hemorrhage (ICH), and cerebral ischemic stroke, are important causes of death and long-term disability worldwide. As an important class of pervasive genes involved in many pathophysiological processes, long non-coding RNAs (lncRNAs) have received attention in the past decades. Multiple studies indicate that lncRNAs are abundant in the CNS and have a key role in brain function as well as many neurological disorders, especially in CNS injuries. Several investigations have deciphered that regulation of lncRNAs exert pro-angiogenesis, anti-apoptosis, and anti-inflammation effects in CNS injury via different molecules and pathways, including microRNA (miRNA), nuclear factor kappa-light-chain-enhancer of activated B cells (NF-κB), phosphatidylinositol-4,5-bisphosphate 3-kinase/protein kinase B (PI3K/AKT), Notch, and p53. Thus, lncRNAs show great promise as molecular targets in CNS injuries. In this article, we provide an updated review of the current state of our knowledge about the relationship between lncRNAs and CNS injuries, highlighting the specific roles of lncRNAs in CNS injuries.

## Main Text

CNS injuries are one of the leading causes of disability and death in modern society, resulting in high medical costs.^1^ CNS injuries usually include traumatic brain injury (TBI), subarachnoid hemorrhage (SAH), intracerebral hemorrhage (ICH), and cerebral ischemic stroke.^2^ The pathological processes of CNS injuries include a series of neurological events, such as inflammation, oxidative stress, apoptosis, autophagy, and blood-brain barrier (BBB) disruption, and eventually result in neuronal cell death in the brain.^3^ Despite the efforts on searching effective methods, patients suffering with severe CNS injuries always end up with poor prognosis. Therefore, new and effective strategies of treatment are urgently needed to reduce the heavy disease and economic burden.

**Figure 1:**
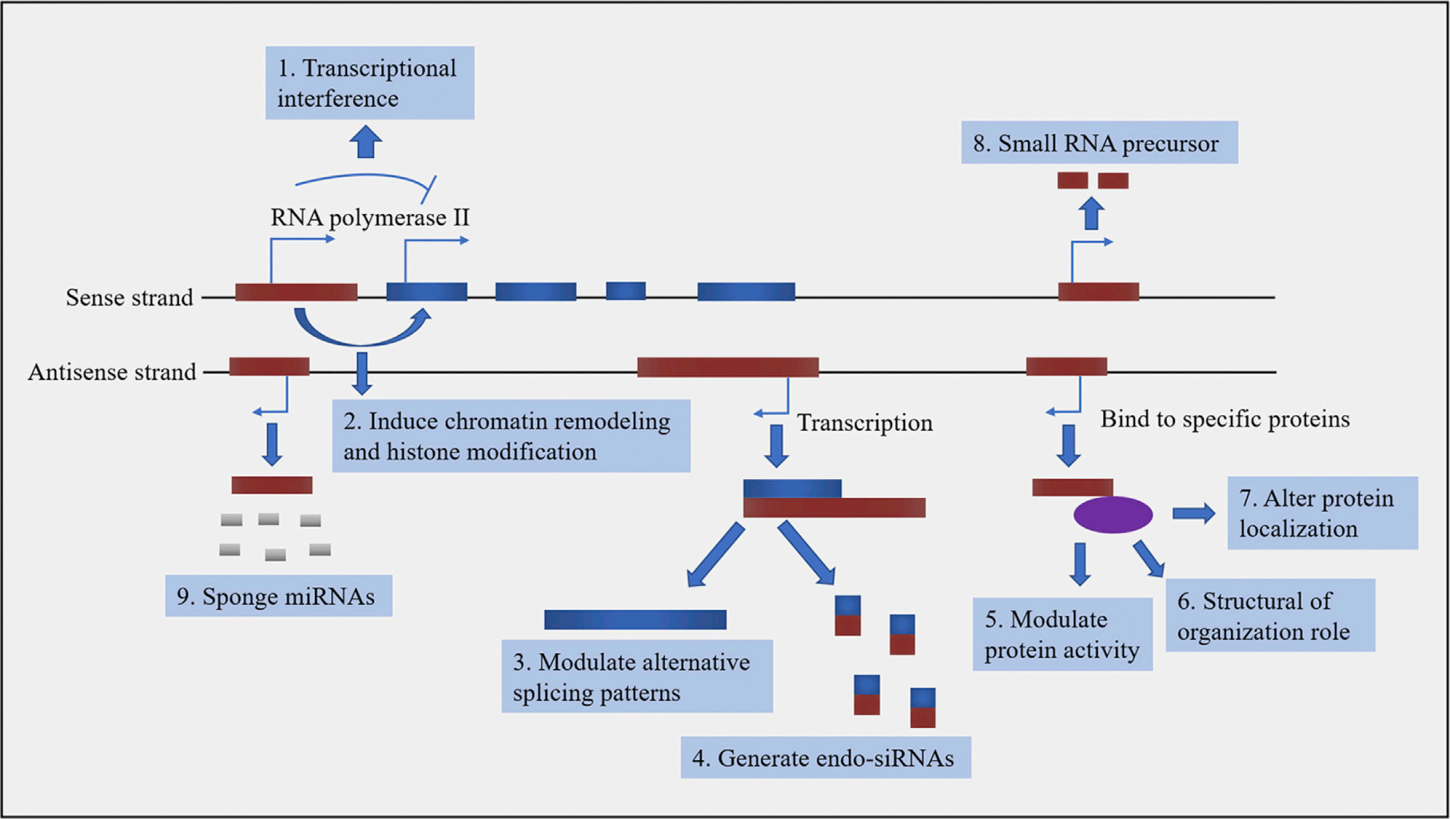
Paradigm for the Functions of lncRNAs lncRNAs (red-brown) can negatively or positively regulate the expression of the coding gene (navy blue) by transcriptional interference (1) or by inducing chromatin remodeling and histone modifications (2). Moreover, transcription of lncRNAs by antisense transcripts can hybridize to their specific coding gene (navy blue), generating alternative splicing (3), or various endo-siRNAs (4). Furthermore, by interacting with specific proteins (purple), lncRNAs can modulate protein activity (5), form cellular substructures (6), or alter protein localization (7). In addition, lncRNAs can be processed to produce small RNAs, such as miRNAs (8). Finally, as miRNA sponges, lncRNAs can influence the endogenous RNAs.

Long non-coding RNAs (lncRNAs) are known as non-coding RNA transcripts greater than 200 nucleotides.^4^ Although lncRNAs were primarily considered as simply transcriptional by-products, accumulated evidence suggests that lncRNAs participate in regulation of various physiological and pathophysiological processes, such as immunity, inflammation, cell differentiation, proliferation, and survival, by modulating the stability and nuclear retention of their target genes.^5^ lncRNAs regulate gene expression at epigenetic, transcriptional, post-transcriptional, and chromatin remodeling levels,^6^, ^7^ and they can activate or inhibit the expression of target genes by directly binding to the target genes or recruiting transcription factors (Figure 1).^8^ Studies have shown that the dysregulation of lncRNAs was implicated in many human diseases.^9^, ^10^, ^11^, ^12^, ^13^ Moreover, the expression profiles of lncRNAs were associated with specific neuroanatomical regions, cell types, and developmental processes, suggesting a potential functional role of lncRNAs in the nervous system.^14^, ^15^, ^16^ The altered expression of lncRNAs was involved in brain development and functional diversification and contributed to diverse neurological disorders such as CNS injuries.^17^, ^18^ In addition, many gain- and loss-of-function approaches found that lncRNAs played an important role in CNS injury-induced secondary brain damage.^19^ In this regard, highlighting the potentially functional roles of lncRNAs in CNS injuries is important. In the present study, we provide an overview of lncRNA functions in CNS injuries and the associated molecular mechanisms.

### lncRNAs in CNS Injuries

During studies assessing the functions of lncRNAs, it was revealed that lncRNAs may act as potential onco- or tumor-suppressor RNAs in numerous cancer types.^20^ This observation led to investigations into the potential role of lncRNAs in various models. Recently, the roles of lncRNAs in CNS injuries were elucidated. Numbers of aberrantly expressed lncRNAs were identified using techniques such as microarray or RNA sequencing (RNA-seq).^21^, ^22^ Specifically, lncRNAs such as metastasis associated lung adenocarcinoma transcript 1 (MALAT1), maternally expressed gene 3 (MEG3), brain-derived neurotrophic factor antisense RNA (BDNF-AS), nuclear enriched abundant transcript 1 (NEAT1), growth arrest-specific transcript 5 (GAS5), and CAMK2D-associated transcript 1 (C2dat1) were found to affect secondary brain injury in CNS injury models (Table 1).

**Table 1:** The Functions and Molecular Targets of lncRNAs in CNS Injuries

lncRNAs	Models	Animals and/or Cells	Expression	Beneficial Functions of the Regulation of lncRNAs	Molecular Targets
ANRIL	MACO	rats, HUVECs	increased	promote angiogenesis, decrease infarction and inflammation	NF-κB
BDNF-AS	H/R injury	HCNs, human astrocytes	increased	increase MMP, ameliorate apoptosis	PI3K/AKT
C2dat1	I/R injury	mice, mouse neurons	increased	promote neuronal survival, decrease inflammation	NF-κB
FosDT	MCAO	rats	increased	ameliorate motor deficits, reduce infarct volume	REST
GAS5	HIBD	rats, rat neurons	increased	reduce brain infarct size, improve neurological function recovery	miR-23a
Gm4419	TBI	mouse astrocytes	increased	decrease inflammation, improve neurological deficits	miR-4661
	OGD/R injury	rat microglial cells	increased	reduce neuroinflammation	NF-κB
H19	OGD/R injury	mice	increased	attenuate neurological deficits and inflammation	–
	I/R injury	rats, SH-SY5Y cells	increased	decrease cell death and autophagy	–
MALAT1	OGD/R injury	mice, mouse neurons	increased	attenuate autophagy, protect BBB function	miR-30a
		BMECs	increased	promote autophagy, inhibit cell death and apoptosis	miR-26b, PI3K/AKT
MEG3	hypoxic injury	PC12 cells	increased	attenuate cell injury and apoptosis	miR-147
	MCAO	mice, rats, HMECs	increased	ameliorate brain lesion, promote angiogenesis	Notch, miR-181b
	SAH	rats, rat neurons	increased	decrease cell death and apoptosis	PI3K/AKT
	OGD/R injury	HT22 cells	increased	improve neurological function, attenuate apoptosis	miR-181b
N1LR	I/R injury	rats	increased	enhance proliferation, inhibit apoptosis, protect BBB function	p53
	OGD/R injury	N2a cells			
NEAT1	TBI	rats	increased	improve neurological function, reduce cell death	–
NKILA	ICH	rats, rat neurons	decreased	induce autophagy, decrease inflammation	NF-κB
RMST	OGD/R injury	mouse neurons	increased	reduce brain infarct size, improve neurological function	–
	MCAO	mice			
SNHG1	OGD/R injury	BMECs	increased	promote cell survival and angiogenesis	miR-199a
TUG1	MACO	rats	increased	decrease cell apoptosis, improve BBB function	miR-9
	OGD/R injury	rat neurons			

lncRNAs, long non-coding RNAs; ANRIL, antisense non-coding RNA in the INK4 locus; MCAO, middle cerebral artery occlusion; HUVECs, human umbilical vein endothelial cells; NF-κB, nuclear factor kappa-light-chain-enhancer of activated B cells; BDNF-AS, brain-derived neurotrophic factor antisense RNA; H/R, hypoxia-reoxygenation; HCNs, human cortical neurons; MMP, mitochondrial membrane potential; PI3K/AKT, phosphatidylinositol-4,5-bisphosphate 3-kinase/protein kinase B; C2dat1, CaMK2D-associated transcript 1; I/R, ischemia-reperfusion; FosDT, Fos downstream transcript; GAS5, growth arrest-specific transcript 5; HIBD, hypoxic/ischemic brain damage; miRNAs, microRNAs; OGD/R, oxygen-glucose deprivation/reoxygenation; TBI, traumatic brain injury; MALAT1, metastasis-associate lung adenocarcinoma transcript 1; BMECs, brain microvascular endothelial cells; MEG3, maternally expressed gene 3; HMECs, human microvascular endothelial cells; SAH, subarachnoid hemorrhage; NEAT1, nuclear enriched abundant transcript 1; ICH, intracerebral hemorrhage; RMST, rhabdomyosarcoma 2-associated transcript; SNHG1, small nucleolar RNA host gene 1; TUG1, taurine-upregulated gene 1.

#### MALAT1

MALAT1, a well conserved, stable, and abundant lncRNA (6.5 kb), was initially considered to be upregulated in solid tumors and associated with cancer cell proliferation, metastasis, survival, and recurrence.^23^ However, growing evidence indicates that MALAT1 has a special role in regeneration after brain injury. It was abundantly expressed in vascular endothelial cells, skeletal muscle, and cardiomyocytes and participated in the pathological inflammatory processes, proliferation, differentiation, myogenesis, and angiogenesis.^24^ It has been shown that MALAT1 could protect brain microvascular endothelial cells (BMECs) from injury caused by oxygen-glucose deprivation-reoxygenation by serving as an autophagy inducer.^25^ In addition, the role of MALAT1 in endothelial cell damage and hyperglycemia-induced inflammation has been previously reported.^26^

#### MEG3

MEG3 is an ∼1.6 kb imprinted gene belonging to the imprinted DLK1-MEG3 locus located at chromosome 14q32.3 DLK1 locus in humans. There are 12 different MEG3 gene transcripts generated by alternative splicing, and the MEG3 gene encodes a non-coding RNA of approximately 1700 nucleotides.^27^ MEG3 was first identified as a lncRNA with the function of a tumor suppressor.^28^ Subsequent results revealed that MEG3 may be involved in cellular remodeling and necessary for the neurons to resist injuries.^29^ Altered expression of MEG3 was observed to mediate ischemic neuronal death by activating p53 both *in vitro* and *in vivo*.^30^ Moreover, downregulation of MEG3 could protect against ischemic damage and enhance neurobehavioral outcomes.^29^ Furthermore, MEG3 functioned as a competing endogenous RNA for miR-181b to regulate 12/15-LOX expression in middle cerebral artery occlusion-induced ischemic infarct of brain nerve cells.^31^

#### BDNF-AS

BDNF is a member of the neurotrophin family of growth factors. It is abundantly expressed during embryonic development and contributes to the development of the nervous system by synchronizing neuronal and glial maturation as well as participating in axonal and dendritic differentiation.^32^ In the brain, BDNF acts on neurons in the hippocampus, cortex, and basal forebrain—areas vital to learning, memory, and higher thinking, supporting neuronal growth, differentiation, plasticity, and survival.^33^ Thus, upregulation of BDNF is thought to have beneficial effects on neurological disorders.^34^ BDNF-AS is one type of lncRNA transcribed by RNA polymerase II without an open reading frame.^35^ Chromatin immunoprecipitation results showed that BDNF-AS reduced the localization of EZH2 and H3K27me3 in the BDNF promoter region and inhibited the expression of BDNF, thereby affecting the growth and differentiation of neural cells. Inhibition of BDNF-AS led to increased protein levels of BDNF and induced neuronal growth and differentiation.^35^ Besides, BDNF-AS was identified to be significantly upregulated in patients with cerebral infarction, whereas BDNF-AS small interfering RNA (siRNA) suppressed hypoxia-reoxygenation (H/R)-induced neurotoxicity through activation of the BDNF.^36^ In addition, a recent study has declared that BDNF-AS knockdown was a novel method to prevent neurotoxicity in mouse embryonic neural stem cell (ESC)-derived neurons.^37^

#### NEAT1

NEAT1 is an ∼3.2 kb novel nuclear lncRNA. It localizes to specific nuclear structures called paraspeckles and is essential for the formation and maintenance of paraspeckles.^38^ Paraspeckles are formed by the binding of paraspeckle protein (PSP)1, PSP2, and p54nrb to the NEAT1 transcriptional start site. The functions of paraspeckles are to mediate the development of corpus luteum and mammary gland as well as modulate cytoplasmic proteins and RNA functions by migrating to the cytoplasm.^39^ Recently, NEAT1 has been proposed to be distributed in many subcellular regions where no paraspeckles were observed,^40^ suggesting that NEAT1 may have other functions besides paraspeckle formation. The effects of NEAT1 on brain injuries were also investigated. For example, it has been shown that NEAT1 was downregulated in cortical neurons in response to neuronal activity.^41^ Moreover, NEAT1 promoted brain injury in septic mice by positively regulating nuclear factor kappa-light-chain-enhancer of activated B cells (NF-κB), while si-NEAT1 transfection could reduce this injury.^42^ In addition, NEAT1 has been shown to play a role in protecting cells subjected to lethal harm from undergoing early apoptosis after TBI, suggesting that NEAT1 potentially exerted a regulatory effect on cell apoptosis.^43^

#### GAS5

GAS5 is a lncRNA that hosts a number of small nucleolar RNAs (snoRNAs) within its introns. So it was originally considered that the biological functions of GAS5 were mediated by introns.^20^ However, recent studies indicate that GAS5 was increased during growth arrest induced by serum starvation or the lack of growth factors.^44^ Moreover, upregulation of GAS5 could cause a slower cell cycle and an increase in cell apoptosis.^45^ These results suggeste that GAS5 plays a key role in normal growth arrest and apoptosis. Therefore, GAS5 may also participate in numerous novel and unexpected cellular functions without introns. Recently, the functions of GAS5 in the neural system were discovered. In a previous study, highly expressed GAS5 was observed in rat hippocampus after TBI by microarray and quantitative real-time PCR analysis, suggesting that GAS5 might be involved in TBI pathological processes.^46^ Further studies confirmed the pro-apoptotic effect of GAS5 by downregulation of miR-335 and upregulation of Rasa1 in a TBI cell model.^47^ Besides, GAS5 was shown to be a promising therapeutic target for the treatment of hypoxic/ischemic brain damage (HIBD).^48^

#### C2dat1

C2dat1 is a sense lncRNA that overlaps with introns 13–15 and exon 14 of the CaMK2D gene in the genome.^49^ It was first discovered in a lncRNA array analysis of a rat middle cerebral artery occlusion (MCAO) model in 2016.^49^ C2dat1 was mainly located in the nucleus of N2a cells, and the inhibition of C2dat1 led to suppression of CaMK2D mRNA and protein.^50^ These results indicated that C2dat1 may interact with CaMK2D directly. It has been shown that C2dat1 was upregulated in murine ischemia/reperfusion (I/R) models and in mouse neuronal cells upon ischemia oxygen-glucose deprivation/reoxygenation (OGD/R). C2dat1 could regulate the expression of CAMK2D/CaMKIIδ in response to OGD/R and C2dat1-induced CaMKIIδ expression, further promoting neuronal survival by activating the NF-κB signaling pathway.^49^

#### Other lncRNAs in CNS Injuries

Besides the six well-studied lncRNAs, there were also other lncRNAs that have been explored in CNS injury models, such as BC048612,^51^ Uc.173,^52^ Gm4419,^53^ HOTAIR,^54^ N1LR,^55^ and RMST.^56^ All these lncRNAs played important roles in CNS injuries by affecting secondary brain damage.

### The Function of lncRNAs in CNS Injuries

Regulation of lncRNA NEAT1, and MALAT1 was first reported to exhibit neuroprotection on CNS injuries in 2014.^57^ Subsequently, a large number of studies have demonstrated that regulation of lncRNAs played a protective role in CNS injuries. The neuroprotection of lncRNAs was attributed to their improvement of cognitive function, promotion of angiogenesis, inhibition of apoptosis and inflammation, regulation of autophagy, and protection of BBB function (Table 2).

**Table 2:** Mechanisms of Regulation of lncRNAs in CNS Injuries

Mechanisms	Factors	Associated Molecules
Improve cognitive function	reduce neuronal loss in the hippocampus and cortex	–
Promote angiogenesis	induce endothelial proliferation, migration and augment vasopermeability	VEGF
Decrease apoptosis	reduce cellular blebbing, chromosomal DNA fragmentation, and formation of apoptotic bodies	p53, Bcl-2, Bax, caspase-3
Suppress inflammation	decrease inflammatory factors and attenuate inflammatory response	NF-κB, TNF-α, IL-1β, IL-6, ICAM-1
Affect autophagy	increase the expression of LC3-II and promote the formation of autophagosome	Beclin-1, LC3
Preserve BBB function	reduce endothelial cell markers and tight junction protein loss	GSTα3, GPx

lncRNAs, long non-coding RNAs; VEGF, vascular endothelial growth factor; Bcl-2, B cell lymphoma-2; Bax, Bcl-2-associated X protein; NF-κB, nuclear factor kappa-light-chain-enhancer of activated B cells; TNF-α, tumor necrosis factor-α; IL-1β, interleukin-1β; IL-6, interleukin-6; ICAM-1, intercellular adhesion molecule 1; LC3, microtubule-associated protein light chain 3; BBB, blood-brain barrier; GSTα3, glutathione S transferase alpha 3; GPx, glutathione peroxidase.

#### Cognitive Function

Cognitive function impairment is a critical factor involved in the pathogenesis of CNS injuries, which may lead to defects in memory, attention, language, executive function, and social communication.^58^, ^59^, ^60^, ^61^ Cognitive function impairment is related to poor long-term outcome in the areas of independent living, return-to-work, and community integration.^62^ Thus, treatment of cognitive function impairment is logical therapeutic targets, and could significantly improve recovery rates and decrease sequelae.

The effects of lncRNAs on cognition after CNS injuries have been explored by many studies. Previous reports have indicated that intracerebroventricular injection of RMST short hairpin RNA (shRNA) significantly decreased brain RMST expression, improved neurological function, and reduced brain infarct size in a mouse MCAO-induced ischemic stroke model.^56^ Furthermore, treatment with exosomes containing lncRNA MALAT1 significantly recovered the function of motor behavior in a rat TBI model.^63^ In addition, it has been shown that knockdown of lncRNA Fos downstream transcript (FosDT) improved the motor recovery in a rat ischemic brain injury model, as measured by rotarod test, beam walk test, and adhesive removal test.^64^ The precise mechanisms underlying how lncRNAs regulated cognitive function were unclear. It has been well established that cognitive dysfunction involved selective neuronal loss in the hippocampus and cortex.^65^ Therefore, regulation of lncRNAs may also improve cognitive function by intervening with these pathological processes. However, this is just our hypothesis, and further studies are needed to explain it.

#### Angiogenesis

Angiogenesis is defined as the formation of new blood vessels from pre-existing vessels.^66^ Angiogenesis plays an important role in mediating functional recovery after CNS injuries, and various pharmacological drugs have been shown to improve functional outcome after CNS injuries by promoting angiogenesis.^67^ Angiogenesis could supply oxygen and nutrients to brain, alleviate brain ischemia, and accelerate the structural remodeling of injured brain, ultimately promoting the recovery of neurological function.^68^ Therefore, promoting angiogenesis is a therapeutic strategy for the treatment of CNS injuries. Angiogenesis is regulated by many vascular growth factors, including vascular endothelial growth factor (VEGF). VEGF can induce endothelial proliferation and migration and augment vasopermeability, making it a significant target for promoting angiogenesis.^67^

Since angiogenesis is a benefit for the damage of brain tissues after CNS injuries, it can be supposed that regulation of lncRNAs may attenuate brain damage in response to CNS injuries by promoting angiogenesis. Consistent with this hypothesis, Wang et al.^69^ proposed that upregulation of lncRNA SNHG1 promoted the angiogenesis of BMECs by activation of VEGF in an OGD/R-induced ischemic stroke model. In another study, it was shown that knockdown of lncRNA MEG3 increased the formation of blood vessels after focal ischemia, as analyzed by micro-CT scanning, indicating that MEG3 downregulation could promote angiogenesis.^29^ Because angiogenesis is one of the major factors determining nerve function recovery after CNS injuries, facilitating angiogenesis by targeting lncRNAs could be a vital therapeutic strategy.

#### Apoptosis

Apoptosis is a very tightly programmed cell death (PCD) that plays a crucial role in normal cell turnover, proper development, and function of the immune system, hormone-dependent atrophy, embryonic development, and chemical-induced cell death.^70^ Insufficient apoptosis can cause autoimmunity or cancer, while accelerated apoptosis can cause neurodegenerative diseases or ischemic damage.^71^ The characteristic morphology changes of apoptosis cells include blebbing, cell shrinkage, nuclear fragmentation, chromosomal DNA fragmentation, and formation of apoptotic bodies.^72^ In the physiological state, apoptosis is a highly regulated and controlled process that confers advantages during an organism’s life cycle. However, if apoptosis occurs in CNS injuries, it may lead to secondary brain injury and exacerbate the damage of CNS injuries.^73^ The functions of lncRNAs in CNS injury-induced neuronal apoptosis have been studied. The results obtained by Wu et al.^55^ demonstrated that overexpression of lncRNA N1LR significantly reduced neuronal apoptosis and neural cell loss in I/R-induced mouse brains, as evaluated by Annexin V/PI staining. In addition, Liang et al.^74^ showed that, in a rat SAH model, lncRNA MEG3 overexpression upregulated the expressions of Bcl-2-associated X protein (Bax), p53, and cleaved caspase-3 and downregulated the expression of Bcl-2, suggesting that MEG3 could increase cell apoptosis. In another study, conducted by Zhong et al.,^75^ it was found that inhibition of lncRNA BDNF-AS was associated with decreased apoptosis in the brain following ischemic injury, as measured by terminal deoxynucleotidyl transferase-mediated dUTP nick end labeling (TUNEL) staining. These results manifestly demonstrate that regulation of lncRNAs could reduce neuronal apoptosis in models of CNS injuries.

So far, studies have only examined the role of lncRNAs on apoptosis after CNS injuries in general. However, apoptosis can be divided into two pathways: the mitochondria-dependent pathway (the intrinsic pathway) and the death receptor-dependent pathway (the extrinsic pathway). The mitochondria-dependent pathway is one of the most important pathways of apoptosis, which is regulated by the B cell lymphoma-2 (Bcl-2) family proteins, including pro-apoptotic proteins (e.g., Bax, Bak, Bid, and Bad) and anti-apoptotic proteins (e.g., Bcl-2, Bcl-x, Bcl-xL, and Bcl-w). Mitochondrial damage induces the release of cytochrome *c* from mitochondrion through alteration of mitochondrial membrane permeability, resulting in the activation of caspase-3 and subsequent apoptosis. On the contrary, the death receptor-dependent pathway is initiated by the binding of Fas ligand to Fas receptor and the binding of tumor necrosis factor (TNF) ligand to TNF receptor, resulting in the activation of caspase-8. Once caspase-8 is activated, the execution phase of apoptosis is triggered.^76^ Therefore, the apoptotic pathway that is associated with the effects of lncRNAs on apoptosis remains unclear and further studies are needed to clarify it.

#### Inflammation

Inflammation plays a critical role in the secondary brain injury after CNS injuries.^77^ Under normal conditions, inflammation is a cellular and molecular process that aims to combat invading pathogens, restore damaged cells, and preserve the health of the tissue.^78^ However, under pathological conditions such as CNS injuries, excessive inflammation could function as a reactionary system to further aggravate brain damage, leading to BBB breakdown, cerebral edema, microglial and astrocytic activation, peripheral leukocyte migration, recruitment, and the release of inflammatory cytokines, such as TNF-α/β, interleukin-1β (IL-1β), -6, -10, and intercellular adhesion molecule 1 (ICAM-1).^79^ These cytokines, in turn, recruit additional blood-borne neutrophils and monocytes into the injured brain, thus expanding the inflammatory cascade and exacerbating the brain injury.^80^

Numerous reports have demonstrated that lncRNAs exert a central effect in inflammation caused by CNS injuries. The role of lncRNAs in CNS injury-induced inflammation was first described by Zhang et al.^81^ in 2017. They found that the pro-inflammatory factors such as E-selectin, MCP-1, and IL-6 were significantly increased in an *in vivo* ischemic stroke model. Silencing of lncRNA MALAT1 by GapmeR further increased the levels of these pro-inflammatory factors, suggesting that MALAT1 has anti-inflammatory effects in ischemic stroke.^81^ Furthermore, in an *in vitro* model of ischemic stroke, high levels of lncRNA Gm4419 elevated the expression of TNF-α, IL-1β, and IL-6, resulting in more aggressive inflammation in astrocytes following OGD/R damage.^53^ In addition, in TBI models, overexpression of lncRNA NEAT1 reduced the production of IL-1β, TNF-α, and nitrite both *in vivo* and *in vitro*, suggesting that NEAT1 had an anti-inflammatory effect.^82^ The underlying mechanisms of the lncRNA-mediated inflammatory reaction are complicated. Studies have indicated that the NF-κB might be the key target, and we will discuss the detailed mechanisms in the following sections. Since CNS injury-triggered inflammatory reaction causes damage not only in brain, but also in other organs, lncRNAs may be a promising target for anti-inflammatory therapy after CNS injuries.

#### Autophagy

Autophagy is a self-catabolic process whereby cells remove cytosolic proteins and organelles and degrade themselves in a stressed or nutrient-deprived state.^83^ During autophagy, the double membrane vesicle, known as autophagosome, first encloses cytoplasmic constituents such as damaged mitochondrion, endosomes, and proteins. Autophagosome then fuses with lysosome to form autolysosome, and the cytoplasmic components are degraded or recycled by acidic lysosomal hydrolases. The process of cargo delivery by autophagosome and degradation by autolysosome is named autophagy flux.^84^ One of the most important functions of autophagy is to maintain the metabolism essential for cell survival in nutrient deprivation or other stress situations. However, dysfunction of autophagy is involved in multiple diseases, including CNS injuries, and extensive activation of autophagy can lead to cell death (type II PCD).^85^, ^86^ Indeed, the role of autophagy in CNS injuries is indefinite; inhibition of autophagy can either attenuate or promote brain damage. Luo et al.^87^ found that suppression of autophagy by 3-methyladenine (3-MA) or bafliomycin A1 (BafA1) reduced TBI-induced cell death and apoptosis, indicating that autophagy promoted apoptosis in TBI. Conversely, Wang et al.^88^ reported that inhibition of autophagy by 3-MA increased brain edema and BBB disfunction and aggravated neurological deficits in SAH, suggesting that autophagy played a beneficial role in SAH.

There were also studies demonstrating that regulation of lncRNAs could affect autophagy in CNS injuries. However, the roles of lncRNA-modulated autophagy in CNS injuries, especially in cerebral ischemic stroke, were also controversial. Li et al.^25^ have shown that upregulation of lncRNA MALAT1 protected BMECs against OGD/R-induced BMEC death by activation of autophagy, suggesting a protective role of MALAT1 and autophagy in ischemic stroke. Interestingly, in another study conducted by Guo et al.,^89^ they revealed that downregulation of lncRNA MALAT1 attenuated OGD/R-induced neuronal cell death by suppression of autophagy, suggesting a detrimental role of MALAT1 and autophagy in ischemic stroke. The discrepancies may be due to the different cell types used in these two studies. Although the lncRNA and ischemic stroke model were the same, the cells used in the Li et al.^25^ experiments were BMECs, while the cells used in Guo et al.^89^ experiments were cerebral cortex neurons. In conclusion, in combination with the previous reports, we thought that, depending on different CNS injury models and cell types, the lncRNAs, autophagy and cell death may have inhibitory, additive, or even synergistic effects.

#### BBB Function

BBB is a highly specialized, semi-permeable barrier that separates the circulating blood from the brain and extracellular fluid in CNS.^90^ Structurally, BBB is formed by brain endothelial cells with tight junction (TJ), and it allows the passage of water, lipid-soluble molecules, glucose, and amino acids, which are important to brain homeostasis and neural functions.^91^ Disruption of BBB is a key secondary injury process following CNS injuries.^92^, ^93^, ^94^ Many pathological conditions, such as TJ breakdown, endothelial cell death, and decreased coupling between TJ proteins and cytoskeletons, may lead to the damage of BBB.^95^ In an *in vitro* model of cerebral ischemic stroke, Li et al.^25^ found that lncRNA MALAT1 protected BBB against OGD/R -induced injury by promoting the survival of BMECs. Furthermore, another *in vitro* study confirmed the protective effects of lncRNA on BBB in ischemic stroke.^96^

### Downstream Molecules of lncRNAs in CNS Injuries

**Figure 2:**
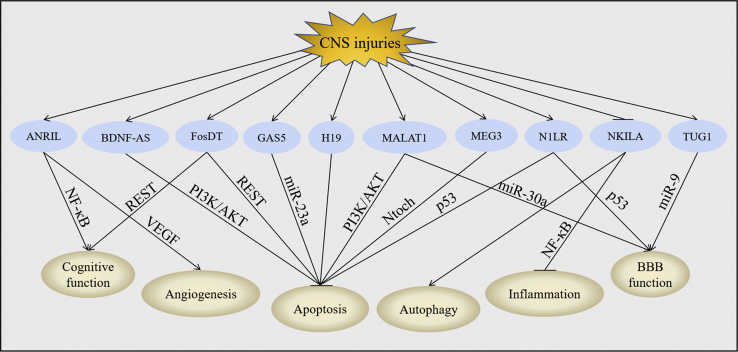
Downstream Molecules of lncRNAs in CNS Injuries CNS injuries upregulated the expression of ANRIL, BDNF-AS, FosDT, GAS5, H19, MALAT1, MEG3, N1LR, and TUG1, but they downregulated the expression of NKILA. Changes of these lncRNAs further leads to the modulation of miRNAs, activation of VEGF and Notch, and inhibition of NF-κB, REST, PI3K/AKT, and p53. Regulation of these downstream molecules subsequently improved cognitive function, promoted angiogenesis, activated autophagy, protected BBB function, and suppressed apoptosis and inflammation post-CNS injuries.

The specific mechanisms mediating the functions of lncRNAs in CNS injuries have yet to be fully explained; a number of downstream molecules of lncRNAs have been proposed that may illustrate their biological effects (Figure 2).

#### MicroRNA (miRNA)

miRNA is a small non-coding RNA molecule approximately 22 nucleotides in length.^97^ Briefly, the formation of a miRNA begins with the transcription of a primary miRNA (pri-miRNA) that harbors the microRNA. The pri-miRNA is then processed by a microprocessor complex to form a precursor miRNA (pre-miRNAs) in the nucleus. The pre-miRNA is then transported into the cytoplasm from the nucleus by exportin-5. In the cytoplasm, the pre-miRNA is cleaved to a single-stranded miRNA by the RNase III-like endonuclease Dicer. Subsequently, each stranded miRNA is incorporated into an Argonaute protein containing RNA-induced silencing complex (RISC). The RISC-loaded miRNA contains a seed region that recognizes complementary sequences at the 3′ untranslated region (UTR) of mRNA, resulting in negative regulation, such as transcript degradation or post-translational suppression.^98^, ^99^, ^100^

miRNAs are present and stable in many mammalian cell types and appear to target nearly 60% of genes in humans.^97^ They control multiple cellular processes, including cell proliferation and differentiation, cell survival, immune responses, angiogenesis, and inflammation.^101^ Therefore, due to the simple structure and the ability to modulate cellular functions, miRNAs have raised the research climax for developing potential miRNA-based therapies. An expanding body of evidence has revealed that lncRNAs could regulate the expression and function of miRNAs in CNS injuries. It has been shown that silencing of lncRNA GAS5 protected against hypoxia/ischemia-induced neonatal brain injury by activation of miR-23a.^48^ Moreover, lncRNA MALAT1 could serve as an endogenous sponge to downregulate miR-26b expression by directly binding to miR-26b, thus promoting BMEC survival under the OGD/R condition.^25^ Furthermore, downregulation of lncRNA MEG3 decreased apoptosis of neural cells after hypoxic damage by targeting miR-147.^96^

How lncRNAs regulate miRNAs has been fully clarified. lncRNAs may act as miRNA sponges or antagomirs, also known as competing endogenous RNAs (ceRNAs), to identify cytoplasmic miRNAs and sequester miRNAs away from mRNAs, thereby regulating the expression of the miRNA target protein. In addition to their function as ceRNAs, lncRNAs also compete with miRNAs for binding to target mRNAs. Furthermore, some lncRNAs are processed to generate certain miRNAs. Finally, lncRNAs can act as platforms for transportation of miRNAs to mRNAs, and even as platforms for transporting outside of cells or between cells.^100^, ^102^, ^103^, ^104^

#### NF-κB

NF-κB is a family of transcription factors that play important roles in inflammation.^105^ NF-κB is abundant in the brain neurons and exhibits diverse functions. It can protect neurons against injuries and regulate neuron inflammation by controlling the transcription of genes such as cytokines, chemokines, and inflammatory transcription factors.^106^ Several CNS diseases are associated with NF-κB activated by inflammatory mediators. A number of negative mediators have been proposed to regulate NF-κB. Among them, the IkB is considered a major brake in NF-kB signaling.^107^ Under physiological conditions, NF-κB is inactive and sequestered in the cytoplasm by binding to its inhibitory kinase IkB. However, under pathologic conditions, such as CNS injuries, IkB can be phosphorylated by IkB kinases (IKKs), leading to the degradation of IkB and subsequent activation of NF-κB.^108^ Activation of NF-κB has been shown to be central to the pathophysiology of inflammatory response triggered by lncRNAs. In ischemic stroke models, the inflammatory response reflexed by the levels of TNF-α, IL-1β, IL-6, and their mediator, NF-κB, were suppressed by knockdown of lncRNA Gm4419^53^ or C2dat1.^49^ Furthermore, in a rat ICH model, the activity of NF-κB and the expression of pro-inflammatory cytokines were much more prominent in lncRNA NKILA knockdown groups than that in sham groups.^109^ Therefore, lncRNAs are significant for the regulation of neuroinflammation in response to CNS injuries by activation of NF-κB in the brain.

How lncRNAs regulate NF-κB has not been well characterized, but there are reports showing that lncRNAs could directly interact with NF-κB or its transcripts. For example, lncRNA NKILA, which locates primarily in the cytoplasm, could inhibit both basal and cytokine-stimulated NF-κB. This inhibition is mediated by the interaction between NKILA and the p65 subunit of NF-κB, leading to the binding of NKILA with NF-κB/IkB to form a stable NKILA/NF-κB/IkB complex. Subsequently, the phosphorylation site of IkB is masked, thus suppressing IKK-induced IkB phosphorylation and NF-κB activation.^110^ In addition, some lncRNAs regulated NF-κB by interfering with the signaling components upstream of NF-κB, which affects the phosphorylation of IKK and IkB. For example, knockdown of lncRNA C2dat1 could downregulate CaMKIIδ expression, suppress IKK phosphorylation, and inhibit NF-κB activity.^111^ Besides, knockdown of lncRNA Gm4419 inhibited the phosphorylation of IkB by physically associating with IkB, resulting in decreased nucleus NF-κB levels.^53^

#### Phosphatidylinositol-4,5-Bisphosphate 3-Kinase/Protein Kinase B (PI3K/AKT) Pathway

The PI3K/AKT pathway is a highly conserved, tightly controlled signaling cascade that plays an essential role in the regulation of cell proliferation, differentiation, metabolism, and survival in both physiological and pathological conditions.^112^ The PI3K/AKT pathway is activated by various receptors, including receptor tyrosine kinases (RTKs) and G-protein-coupled receptors (GPCRs). Stimulation of RTKs or GPCRs leads to the activation of class I PI3Ks which phosphorylate phosphatidylinositol (4,5)-bisphosphate (PIP2) to phosphatidylinositol (3,4,5)-trisphosphate (PIP3). Subsequently, PIP3 recruits AKT to the plasma membrane and phosphorylates AKT on its serine 472 site and threonine 308 kinase site. The phosphorylated AKT is involved in its downstream mTORC1-mediated response to biogenesis of ribosomes and proteins.^113^, ^114^, ^115^ This activity can be reversed by the PI3K antagonist phosphatase and tensin homolog (PTEN) through dephosphorylation of PIP3 to PIP2. Notably, the PI3K/AKT pathway has a critical role in neuronal survival in a variety of CNS injuries, and regulation of this pathway has the potential to treat brain injury.^116^

Recently, multiple lncRNAs were found to provide protection in CNS injury models by activation of the PI3K/AKT pathway. Xin et al.^117^ found that overexpression of lncRNA MALAT1 decreased cell apoptosis by activation of PI3K and phosphorylation of AKT in cerebral I/R injury. Furthermore, Zhong et al.^36^ indicated that knockdown of lncRNA BDNF-AS attenuated H/R-induced mitochondrial membrane potential (MMP) decrease and cell apoptosis through the activation of the PI3K/AKT pathway. Moreover, Liang et al.^74^ revealed that lncRNA MEG3 aggravated SAH-induced neuronal cell apoptosis via inhibiting the PI3K/AKT pathway. These data suggest that the PI3K/AKT pathway may be a potential therapeutic target for lncRNAs in CNS injuries.

But how lncRNAs regulate the PI3K/AKT pathway in CNS injuries is uncertain. There have been several explanations, and these explanations are consistently related to miRNA-mediated PTEN. That means lncRNAs first regulate miRNAs, which further modulate PTEN and the downstream PI3K/AKT pathway. For instance, by inhibiting the expression of miR-103, lncRNA GAS5 significantly enhanced the expression of PTEN, which negatively regulated the PI3K/AKT pathway, to promote cancer cell apoptosis.^118^ In another case, lncRNA HULC activated the PI3K/AKT pathway by inhibiting the miR-15a-PTEN axis in liver cancer.^119^ In addition, downregulation of lncRNA fer-1-like family member 4 (FER1L4) could result in a reduction of PTEN expression by freeing miR-106a-5p, thus activating the PI3K/AKT pathway.^120^ So, by combination with previous literature, it can be supposed that lncRNAs may also mediate the PI3K/AKT pathway by modulation of the miRNA-PTEN axis in CNS injury models. However, this is just our speculation, and further studies are needed to confirm it.

#### Notch Pathway

The Notch signaling pathway belongs to an evolutionarily conserved signal transduction system and plays critical roles in cell proliferation, differentiation, apoptosis, and homeostasis of multicellular organisms. It also influences synaptic plasticity as well as learning and memory in the adult brain.^121^ Binding of Notch receptors to ligands activates Notch intracellular cytoplasmic domain (NICD), which translocates to the nucleus and induces transcription of Notch target genes, such as hairy and enhancer of split-1 (Hes-1) and Hes-related with YRPW motif-1 (Hey-1).^122^ Current evidence has highlighted a key role of the Notch pathway in CNS injuries. The Notch pathway is activated in endothelial cells in response to ischemia and determines the formation of native arterial collateral networks. Inhibition of Notch pathway has been shown to impair reparative angiogenesis after ischemia.^123^ lncRNAs could also facilitate Notch to regulate angiogenesis in ischemic stroke. It has been suggested that the protein levels of NICD, Hes-1, and Hey-1 were decreased by overexpression of MEG3 but increased by knockdown of MEG3 in the ischemic brain. Furthermore, inhibition of Notch by N-[N-(3,5-difluorophenacetyl)-L-alanyl]-S-phenylglycinetbutylester (DAPT) impaired MEG3-induced angiogenic migration and sprouting.^29^

The mechanisms of how lncRNAs regulate Notch pathway have not been explained in detail. It has been shown that lncRNA steroid receptor RNA activator (SRA) could activate the Notch pathway by functioning together with DEAD box protein 5 (Ddx5). Ddx5 localized to recombination signal-binding protein J (RBP-J)-binding sites of the Notch target genes Hes-1 and pre T cell antigen receptor alpha (preTCRα). Upregulation of SRA promoted the activity of Ddx5 and increased the expression of Hes-1 and preTCRα, thereby activating the Notch pathway.^124^ However, the mechanisms by which other lncRNAs regulate the Notch pathway are unclear, which is an interesting aspect worth exploring.

There are four structurally related Notch receptors (Notch1–4) that bind transmembrane ligands of the Jagged (Jagged-1 and Jagged-2) or the Delta-like (DLL1, DLL3, and DLL4) families. Binding of Notch receptors to ligands induces separation of the extracellular receptor subunit from the transmembrane subunit.^125^ Although the Notch receptors are structurally similar, each member appears to exert a specific biological role. Notch1 mutation can cause vascular malformations, Notch2 mutation can cause vascular and renal defects, and Notch3 mutation can cause cerebral autosomal-dominant arteriopathy with subcortical infarcts and leukoencephalopathy (CADASIL) syndrome. Despite that, Notch4 is dispensable for embryonic development; some of its functions overlap with those of Notch1.^126^ Since there has been reports showing that Notch can be regulated by lncRNA MEG3 in ischemic stroke, further studies are needed to confirm whether other lncRNAs could regulate Notch in CNS injury models and which Notch receptor is regulated by lncRNAs.

#### p53

The p53 protein is a pleiotropic transcription factor that plays a significant role in the determination of cell fate under several conditions.^127^ Upon cellular stress, such as DNA damage, oxidative stress, and nerve growth factor (NGF) withdrawal, p53 binds to a consensus response element (p53RE) in the promoter of p53 target genes, such as the pro-apoptotic genes Bax and PUMA, cell-cycle arrest, and DNA-repair gene p21, thus leading to their transcription activation and triggering cell-cycle arrest, promoting apoptosis, regulating differentiation, and altering cellular lifespan.^128^, ^129^, ^130^ In addition, evidence has suggested that p53 contributes to direct neurons toward a specific phenotype in critical conditions, such as during development and following cellular damage. It has been reported that ischemic injury was strongly correlated with an increase in p53 activity^127^ and that the activation of p53 in response to ischemic injury was regulated by lncRNA. Wu et al.^55^ implied that lncRNA N1LR protected the brain from ischemic stroke-induced apoptosis by suppressing p53 phosphorylation. lncRNAs have been shown to regulate p53 by affecting p53 mRNA stability and impacting transcription of p53 target genes. For example, N1LR could serve as an inhibitor to prevent the p53 protein from being phosphorylated on serine 15, thereby inhibiting the activity of p53.^55^

### Other Aspects of lncRNA Research in CNS Injuries

CNS injuries are a diverse group of disorders that involve many characteristics. There are various pathological processes responsible for the secondary damage of CNS injuries, including glutamate excitotoxicity, inflammation, oxidative stress, and apoptosis. Although the functions of lncRNAs on CNS injury-induced cognitive function, oxidative stress, apoptosis, inflammation, autophagy, and BBB disruption have been widely described, its role in oxidative stress and excitotoxicity have not been illustrated so far.

#### Oxidative Stress

Oxidative stress is an event caused by the imbalance between the biological systems, leading to the generation of free radicals such as reactive oxygen species (ROS) and the systems responsible for the removal of free radicals, also known as the enzymatic and non-enzymatic antioxidant cellular defense systems. The excessive generation of free radicals due to excitotoxicity or depletion of the antioxidant system induces toxic effects, protein oxidation, DNA redox imbalance, and mitochondrial electron transport chain (METC) inhibition, resulting in oxidative stress damage.^131^, ^132^ Recently, there has been growing evidence demonstrating that oxidative stress plays a crucial role in the development of cerebral edema, inflammation, and apoptosis and contributes to secondary neuronal damage in CNS injuries.^133^, ^134^, ^135^

There were also studies showing that lncRNAs could regulate oxidative stress. Zhang et al.^136^ indicated that lncRNA FOXD3-AS1 could regulate oxidative stress-induced apoptosis via sponging miRNA-150 in human lung epithelial cells. Moreover, Gao et al.^137^ proposed that lncRNA MT1DP could aggravate cadmium-induced oxidative stress by interaction with miR-365 and suppression of nuclear factor erythroid 2-related factor 2 (Nrf2) in human hepatocellular carcinoma cell line HepG2. In addition, downregulation of lncRNA LINC00963 inhibited oxidative stress by activation of the forkhead box O (FoxO) signaling pathway in chronic renal failure.^138^ Therefore, further studies are needed to estimate whether lncRNAs could affect oxidative stress in CNS injuries.

#### Excitotoxicity

Excitotoxicity occurs early after CNS injuries and plays an important role in apoptosis, necrosis, and autophagy.^139^ Excitotoxicity begins with the abnormal influx of Na^+^ and efflux of K^+^, contributing to the stopping or slowing of ATP production. The decreased ATP levels, in response to Na^+^/K^+^ imbalance, further reduces the reuptake of glutamate, which is the main excitatory neurotransmitter in the brain. Glutamate can mediate excitotoxicity by permitting the Ca^2+^ influx into cells via the activation of N-methyl-D-aspartate receptors (NMDARs), α-amino-3-hydroxy-5-methyl-4-isoxazolepropionic acid receptors (AMPARs), and kainate receptors. Cellular accumulation of Ca^2+^ produces a series of cellular events (excitotoxicity) that lead to mitochondrial failure and apoptosis.^140^, ^141^, ^142^, ^143^ Therefore, glutamate is considered the main contributor that triggers excitotoxic cell damage, and a variety of experimental and clinical studies support the views that CNS injuries induce excitotoxicity by increase of glutamate.^144^, ^145^

The functions of lncRNAs in excitotoxicity have also been well established. It has been shown that silencing of lncRNA BDNF-AS attenuated Aβ_25–35_-induced neurotoxicity in Alzheimer’s disease (AD).^146^ Besides, lncRNA Gadd45a has been suggested to be associated with sevoflurane-induced neurotoxicity in rat neural stem cells.^147^ Furthermore, lncRNA SNHG1 could promote α-synuclein aggregation and excitotoxicity by targeting the miR-15b-5p/SIAH1 axis in Parkinson’s disease (PD).^148^ Thus, it can be speculated that lncRNAs may also participate in CNS injury-induced excitotoxicity. However, further studies are required to verify it.

### Conclusions

lncRNAs play a key role in CNS injuries and are involved in a number of cellular and molecular processes of CNS injuries. In this review, we describe the functions of lncRNAs as well as some downstream moleculars of lncRNAs in CNS injuries. These observations make lncRNAs more attractive therapeutic targets for developing new therapeutic strategies to achieve better outcomes for patients suffering from CNS injuries. It is certain that further studies are needed to clarify the specific mechanisms of lncRNAs in CNS injuries. Finally, targeting lncRNAs may hold promise for clinical therapies.

## Author Contributions

L.Z. conducted the literature review and initial draft of the manuscript. H.W. provided overall supervision and edited the manuscript. Both L.Z. and H.W. read and approved the final manuscript.

## Conflicts of Interest

The authors declare no competing interests.
